# Multi-omics analysis of molecular mechanisms driving the grafting- enhanced resistance of tea plants to *Colletotrichum camelliae*

**DOI:** 10.3389/fpls.2025.1750493

**Published:** 2026-01-14

**Authors:** Yue-Xin Li, Kai-Qin Lin, An-Ran Wang, Jie Wei, Dong-Hai Yan, De-Gang Zhao

**Affiliations:** 1 Tea Research Institute, Guizhou Academy of Agricultural Sciences, Guiyang, China; 2 Guizhou Provincial Government, Guizhou Key Laboratory of Molecular Breeding for Characteristic Horticultural Crops, Guiyang, China; 3 The Key Laboratory of Plant Resources Conservation and Germplasm Innovation in Mountainous Region (Ministry of Education), College of Life Sciences/College of Tea Sciences, Guizhou University, Guiyang, China; 4 Plant Conservation & Breeding Technology Center, Biotechnology Institute of Guizhou Province, Guizhou Academy of Agricultural Sciences, Guiyang, China

**Keywords:** *Camellia sinensis*, *Colletotrichum camelliae*, disease resistance, grafting, multi-omics

## Abstract

**Background:**

The fungal pathogen *Colletotrichum camelliae* causes a devastating disease that severely limits tea plant (*Camellia sinensis*) yield and quality. Grafting onto resistant rootstocks offers a sustainable control strategy, yet resistant rootstocks confer scion protection remains obscure.

**Methods:**

Susceptible cultivar ‘Qianmei 818’ was grafted onto resistant ‘Qianmei 419’. Profiled systemic defenses using integrated RNA-seq, sRNA-seq, and metabolomics, complemented by phytohormone and defense-enzyme assays and qRT-PCR validation.

**Results:**

Hetero-grafting conferred near-complete resistance, reducing lesion diameters by 98.71% compared to ungrafted controls, with elevated PAL activity and accumulation of IAA, GA3, and MeSA. Multi-omics analyses identified 1205 differentially expressed genes, 157 differentially expressed miRNAs, and 791 differential metabolites. Pathway integration indicated extensive reprogramming of phenylpropanoid biosynthesis, sulfur metabolism, and plant hormone signaling. Notably, specific miRNA-mRNA regulatory modules, such as downregulation of csi-miR395b-3p and a novel miR397 paralleled up-regulation of their targets in sulfur assimilation (*CsAPS1*) and lignin biosynthesis (*CsCCoAOMT*, *CsCCR2*), respectively, linking miRNA control to reinforcement of structural and biochemical defenses.

**Conclusions:**

Resistant rootstocks activate scion-wide defense networks through miRNA-mediated transcriptome-metabolome remodeling, achieving robust resistance while maintaining tea quality. The elucidated modules provide actionable targets and genetic resources for breeding and grafting strategies toward sustainable disease management.

## 1 Introduction

*Camellia sinensis*, commonly known as tea plant, is one of the most widely cultivated and economically important crops globally. A prevalent fungal disease caused by *Colletotrichum camelliae* (*C. camelliae*) affects tea plants across major tea-producing regions in China, leading to substantial yield losses and compromised tea quality, posing a significant threat to the sustainable development of the tea industry (Zhang et al., 2024). Although chemical treatments provide short-term disease suppression, the resulting pesticide residues raise significant concerns regarding tea safety and environmental health (Sun et al., 2017). Thus, developing environmentally sustainable disease management strategies and enhancing the inherent resistance of tea plants are imperative for the long-term viability of the tea sector (Chen et al., 2018; Zhao et al., 2023).

Grafting is an established horticultural practice widely employed to improve plant resistance and performance across diverse crops (Goldschmidt, 2014; Kyriacou et al., 2017). In various species, grafting onto resistant rootstocks confers systemic disease resistance to susceptible scions through multiple mechanisms, including the long-distance transport of mobile signals such as hormones (e.g., salicylic acid and jasmonic acid), proteins, mRNAs, and small RNAs (including miRNAs and siRNAs) via the phloem (Guan et al., 2012; Jensen et al., 2012; Jeynes-Cupper and Catoni, 2023). These systemic signals are capable of reprogramming gene expression in the scion, promoting the accumulation of defense-related secondary metabolites, activating systemic acquired resistance, and inducing epigenetic modifications that collectively strengthen the plant’s innate immunity against biotic stress (Spanò et al., 2020). In tea cultivation, mounting evidence demonstrates that judicious selection of rootstocks can effectively enhance scion resistance, yield, and quality (Zhang, 1979; Wang, 2007; Karunakaran and Ilango, 2019). Notably, rootstock grafting can systemically reprogram the metabolic landscape of tea scions, leading to the enhanced accumulation of defense-related secondary metabolites, which are critical components of plant innate immunity against biotic stressors (Deng et al., 2017; Liu et al., 2025). This graft-mediated metabolic reprogramming underscores the potential of utilizing resistant rootstocks as a viable strategy to fortify scions against fungal pathogens such as *C. camelliae*.

Despite these advances, the precise long-distance signal transduction networks—especially the identity, mobility, and regulatory roles of rootstock-derived small RNAs and associated RNA-silencing machinery—in graft-induced resistance to *C. camelliae* remain largely unknown. In preliminary work, leveraging novel cultivars developed by the Guizhou Provincial Tea Research Institute, we established an effective grafting system pairing the *C. camelliae* susceptible cultivar ‘Qianmei 818’ as scion with the resistant cultivar ‘Qianmei 419’ as rootstock. Field trials revealed that grafting resulted in a pronounced reduction in *C. camelliae* incidence in ‘Qianmei 818’ scions while preserving their characteristic high epigallocatechin gallate (EGCG) content. This grafting system thus provides an ideal platform for dissecting the molecular signaling events underlying grafting-induced disease resistance in tea. To further elucidate these intricate molecular mechanisms, the present study implements a comprehensive multi-omics approach, integrating transcriptome (RNA-Seq), metabolome, and small RNA (sRNA-Seq) analyses. This integrated analysis aims to explore the intricate biological interactions and signal transduction pathways occurring between the scion and rootstock, thereby providing critical theoretical insights and practical guidance for enhancing resistance to *C. camelliae* in tea plants.

## 2 Results

### 2.1 Grafting strongly reduces lesion development caused by *C. camelliae*

**Figure 1:**
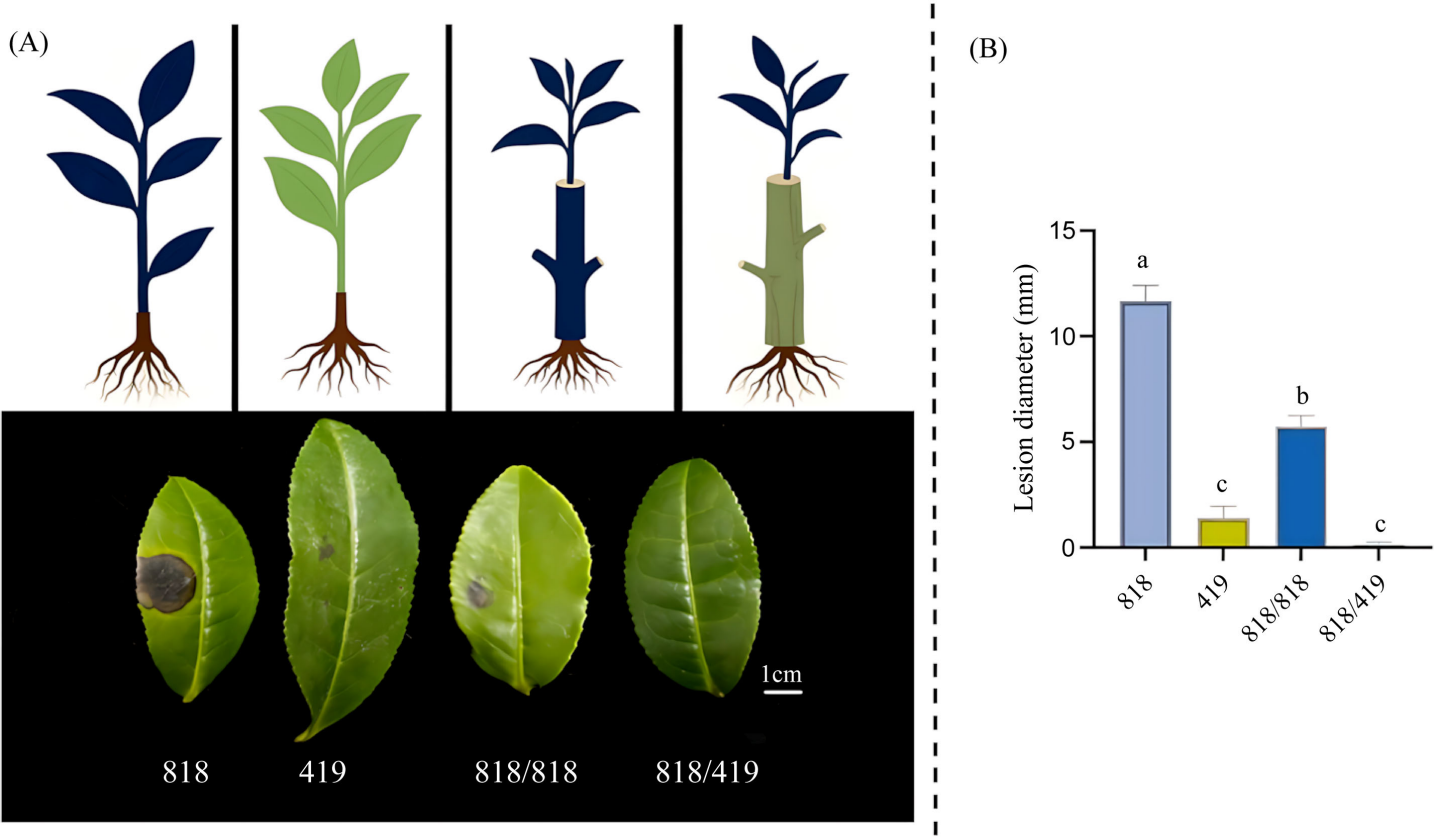
Effect of grafting on the susceptibility of tea plants to *C. camelliae*. **(A)** Symptoms 3 day after inoculation of leaves from four different grafting combinations (Susceptible cultivar ‘818’ alone; resistant cultivar ‘419’ alone; ‘818’ grafted on ‘818’; ‘818’ grafted on ‘419’). The 5-mm-diameter mycelial plugs used for inoculation were removed prior to the examination of the leaves. **(B)** Average lesion diameters 3 days after leaf inoculation. Different letters above the bars indicate significantly different lesion diameters according to Duncan’s multiple comparison test (*p* < 0.05). The error bars represent the standard error of the mean (n=30).

The susceptibility of tea plants to *C. camelliae* was evaluated across different grafting combinations, revealing markedly distinct outcomes. The ungrafted susceptible scion cultivar ‘818’ developed the largest lesion diameter, averaging 11.67 ± 0.74 mm. In contrast, the resistant rootstock ‘419’ demonstrated strong resistance, with lesion diameters limited to 1.13 ± 0.07 mm. Self-grafted ‘818/818’ plants exhibited a significant 50.9% reduction in lesion size compared to ungrafted ‘818’. Remarkably, hetero-grafted ‘818/419’ plants showed an even greater decrease, with lesion diameters reduced by 98.7% relative to ungrafted ‘818’ and by 80.3% compared to ‘818/818’ (all differences statistically significant; p < 0.05) (**Figures 1A, B**). These findings robustly confirm that grafting substantially enhances resistance to *C. camelliae* in the susceptible cultivar, with hetero-grafting onto resistant ‘419’ rootstock conferring superior protective effects. This underscores the potential of resistant rootstocks to modulate scion disease resistance through grafting.

### 2.2 Grafting reshapes the production of defense enzymes, phytohormones, and tea quality-related metabolites

To elucidate the physiological and biochemical bases underpinning grafting-enhanced disease resistance, we systematically analyzed key components in the scion leaves of ‘818’, ‘419’, and grafted plants (‘818/818’ and ‘818/419’).

#### 2.2.1 Defense enzyme activities

**Figure 2:**
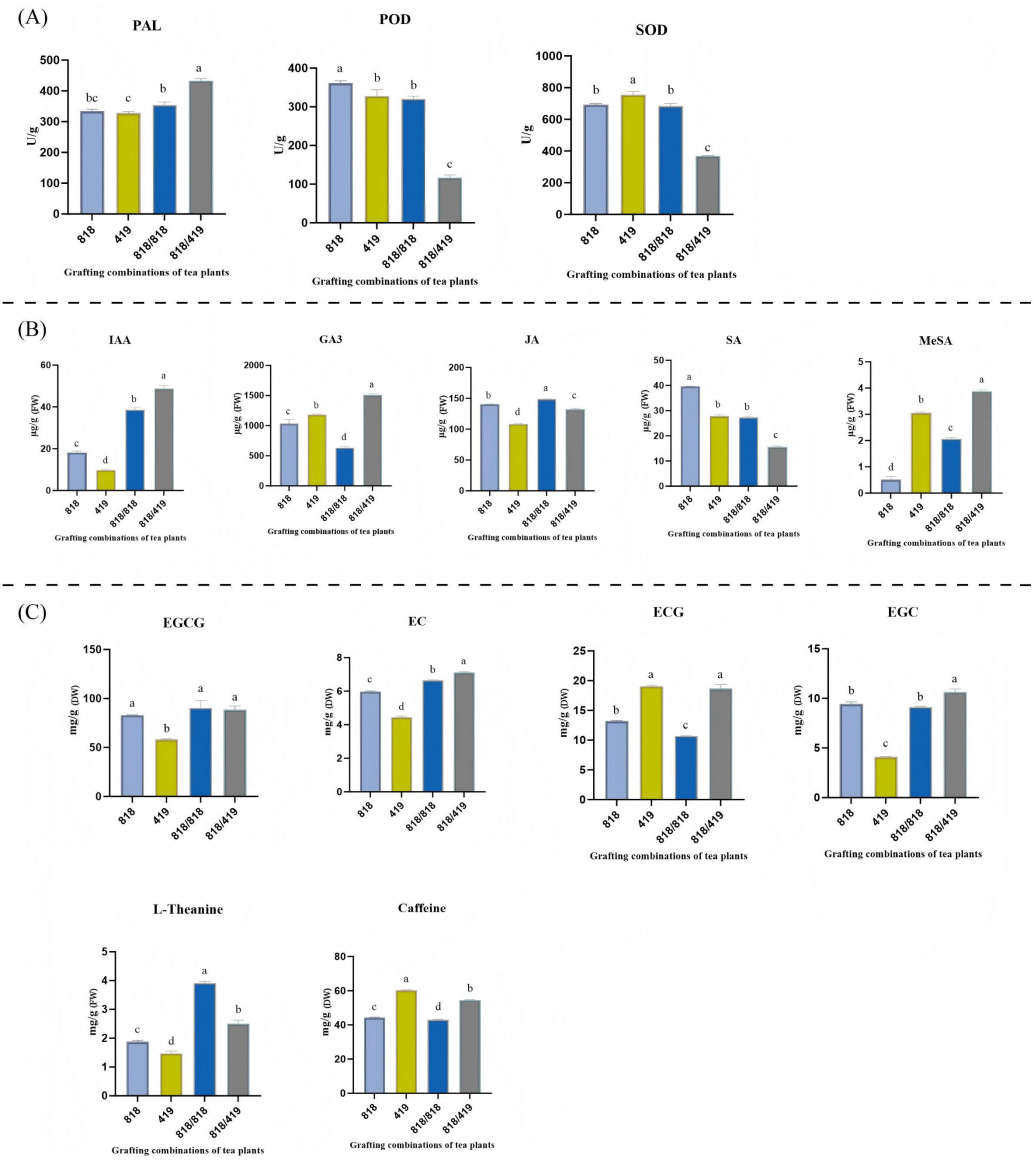
Physiological and biochemical effects of grafting on tea plants. **(A)** Activity of defense-related enzymes phenylalanine ammonia-lyase (PAL), peroxidase (POD) and superoxide dismutase (SOD) in tea leaves (in units per gram of leaf tissue). **(B)** Leaf tissue content in endogenous hormones Indole-3-acetic acid (IAA), gibberellin A3 (GA3), salicylic acid (SA), methyl salicylate (MeSA) and jasmonic acid (JA) (in µg per gram of leaf tissue). **(C)** Leaf tissue content in quality-related compounds epigallocatechin gallate (EGCG), epicatechin (EC), epigallocatechin (EGC), epicatechin gallate (ECG), L-theanine and caffeine (in mg per gram of leaf tissue). For a given compound, different letters above the bars indicate significant differences between grafting treatments according to Duncan’s multiple comparison test (*p* < 0.05). The error bars represent the standard error of the mean (n=3).

Phenylalanine ammonia-lyase (PAL), peroxidase (POD) and superoxide dismutase (SOD) are widely recognized as key biochemical indicators for evaluating disease resistance mechanisms in grafted plants (Singh et al., 2022). In our study, similar patterns were observed for the leaf content in the three enzymes, with statistically significant (*p* < 0.05) and markedly different values for heterografted ‘818/419’and for the three other plant groups (**Figure 2A**). Compared to ungrafted ‘818’, ungrafted ‘419’ and self-grafted ‘818/818’, the values for ‘818/419’ were 18.23-24.29% higher for PAL activity and they were 67.60-63.51% and 51.02-46.64% lower for POD and SOD activities, respectively. These contrasted enzyme activity profiles indicate that hetero-grafting with the resistant rootstock ‘419’ significantly elevates PAL activity but suppresses POD and SOD activity, potentially enhancing phenylpropanoid metabolism and contributing to improved defense responses in tea plants.

#### 2.2.2 Phytohormone profiles

Plant hormones play crucial roles in regulating graft healing and defense responses. We focused on five phytohormones, Indole-3-acetic acid (IAA), gibberellin A3 (GA3), salicylic acid (SA), methyl salicylate (MeSA) and jasmonic acid (JA), because of their established roles in promoting growth, wound healing, and stress defense in grafted plants (Sharma and Zheng, 2019; Zhang et al., 2022; Wang et al., 2020a). Our analysis revealed that hetero-grafting with the resistant rootstock ‘419’ profoundly altered the hormonal landscape in the scion (**Figure 2B**). The hetero-graft ‘818/419’ exhibited significantly elevated levels of IAA and GA_3_, suggesting enhanced growth and wound-healing capacity. Regarding defense-related hormones, a notable shift was observed in the salicylate pathway: while basal SA levels were lower in ‘818/419’, its volatile derivative MeSA accumulated to the highest level. Concurrently, JA content was also reduced in the hetero-graft compared to the susceptible ‘818’ and self-grafted ‘818/818’. These results indicate that the resistant rootstock ‘419’ reprograms scion hormone homeostasis, promoting growth regulators (IAA, GA_3_) and channeling SA into a mobile defense signal (MeSA), while attenuating basal JA and SA pathways.

#### 2.2.3 Quality-related metabolites

The cultivar ‘818’ is appreciated by tea consumers for its high epigallocatechin gallate (EGCG) content. We measured EGCG and other key quality-related compounds such as epicatechin (EC), epigallocatechin (EGC), epicatechin gallate (ECG), L-theanine and caffeine in ‘818’, ‘419’, and grafted plants. Cultivar ‘818’ exhibited significantly higher EGCG, EC, EGC, and L-theanine compared with ‘419’ (*p* < 0.05), whereas ‘419’ contained elevated ECG and caffeine. Self-grafting further increased EGCG, EC, and L-theanine but reduced ECG and caffeine in ‘818/818’. In hetero-grafted ‘818/419’ plants, the EGCG content was similar to levels of ungrafted ‘818’, while those of EC and EGC were significantly higher than in all other groups (*p* < 0.05). L-theanine also increased in ‘818/419’ compared to the ungrafted groups (*p* < 0.05). Meanwhile, ECG and caffeine exceeded both ‘818’ and ‘818/818’ levels (*p* < 0.05) (**Figure 2C**). These results indicate that hetero-grafting integrates key quality traits from both rootstock and scion, synergistically enhancing levels of EC, EGC, and L-theanine while preserving EGCG and enriching ECG and caffeine, thereby improving overall tea quality.

### 2.3 Grafting induces transcriptomic remodeling in the scion

To explore molecular mechanisms of grafting-enhanced disease resistance, transcriptome sequencing was performed on scions from ungrafted ‘818’, self-grafted ‘818/818’, and hetero-grafted ‘818/419’. High-quality data totaled 56.77 Gb, with individual samples yielding ≥5.96 Gb clean reads, Q30>96.6%, and genome mapping rates >85%. Differential expression analysis identified 229 differentially expressed genes (DEGs; 75 upregulated, 154 downregulated) in ‘818/818’ *vs* ‘818’; 565 DEGs (155 upregulated, 410 downregulated) in ‘818/419’ *vs* ‘818/818’; and 1205 DEGs (299 upregulated, 906 downregulated) in ‘818/419’ *vs* ‘818’ (**Supplementary Figure S1A**), indicating stronger regulatory effects in plants grafted on resistant rootstocks.

**Figure 3:**
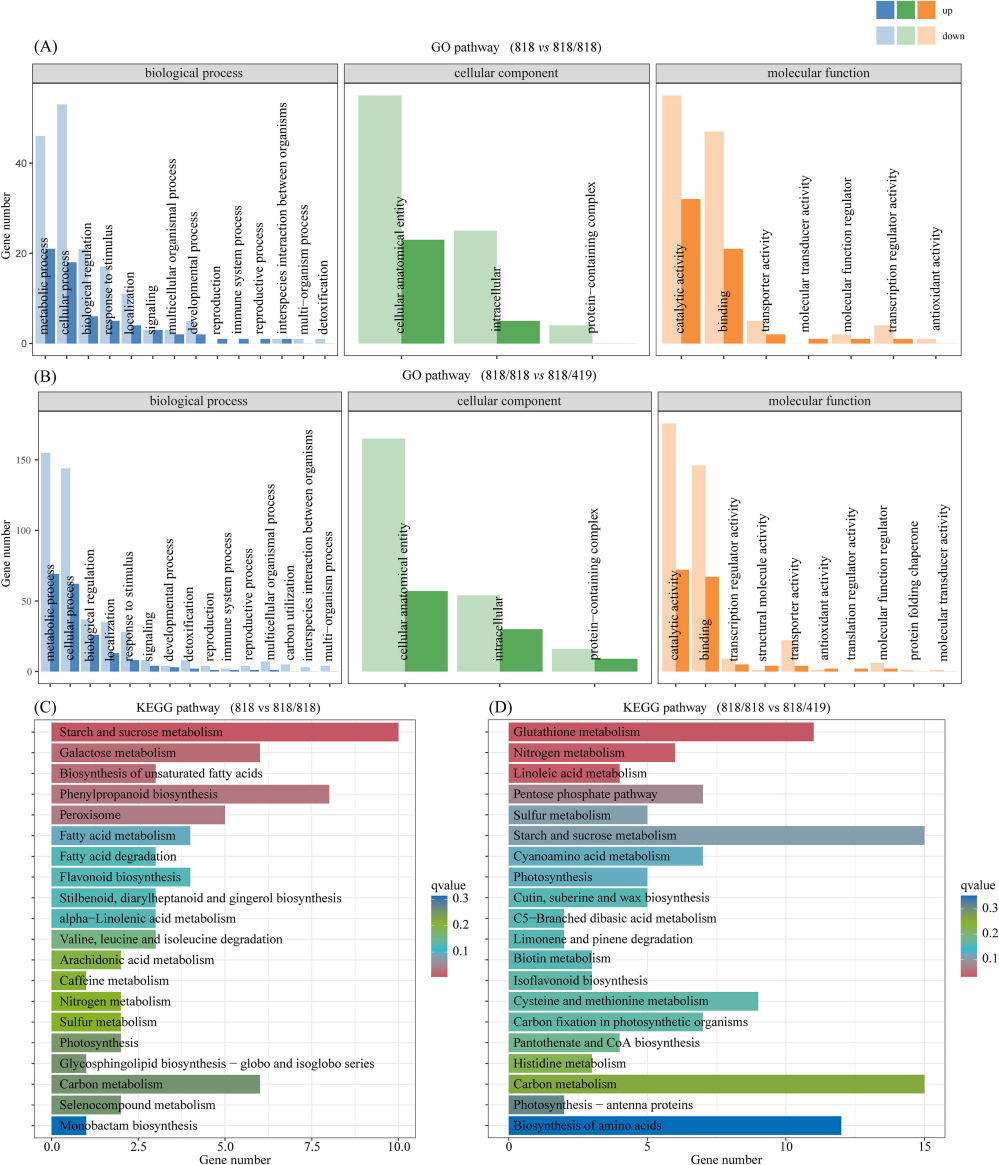
Functional enrichment of DEGs from ‘818 *vs* 818/818’ and ‘818/818 *vs* 818/419’. **(A, B)** Gene Ontology (GO) enrichment; **(C, D)** Kyoto Encyclopedia of Genes and Genomes (KEGG) pathway enrichment.

Gene ontology (GO) enrichment revealed that grafting primarily modulated genes involved in metabolic and cellular processes, cellular components, catalytic activity, and binding, whereas resistant rootstocks broadened activation to include metabolic processes, signal transduction, and transcriptional regulation (**Figures 3A, B**). Kyoto Encyclopedia of Genes and Genomes (KEGG) analysis showed DEGs in ‘818 *vs* 818/818’ enriched pathways such as starch/sucrose metabolism, galactose metabolism, unsaturated fatty acid biosynthesis, phenylpropanoid biosynthesis, and peroxisome function (**Figures 3C, D**). In contrast, ‘818/818 *vs* 818/419’ DEGs were enriched in glutathione, nitrogen, sulfur metabolism, linoleic acid metabolism, pentose phosphate pathway, carbon metabolism, photosynthesis, and stress-response pathways including chloroplast light-harvesting complex assembly and cysteine/methionine metabolism. Thus, grafting mainly induced metabolic remodeling and cellular adaptation facilitating grafting stress tolerance and transport, while resistant rootstocks potentiated defense-related metabolic and signaling pathways in the scion.

### 2.4 Grafting modulates miRNA expression and target pathways

**Figure 4:**
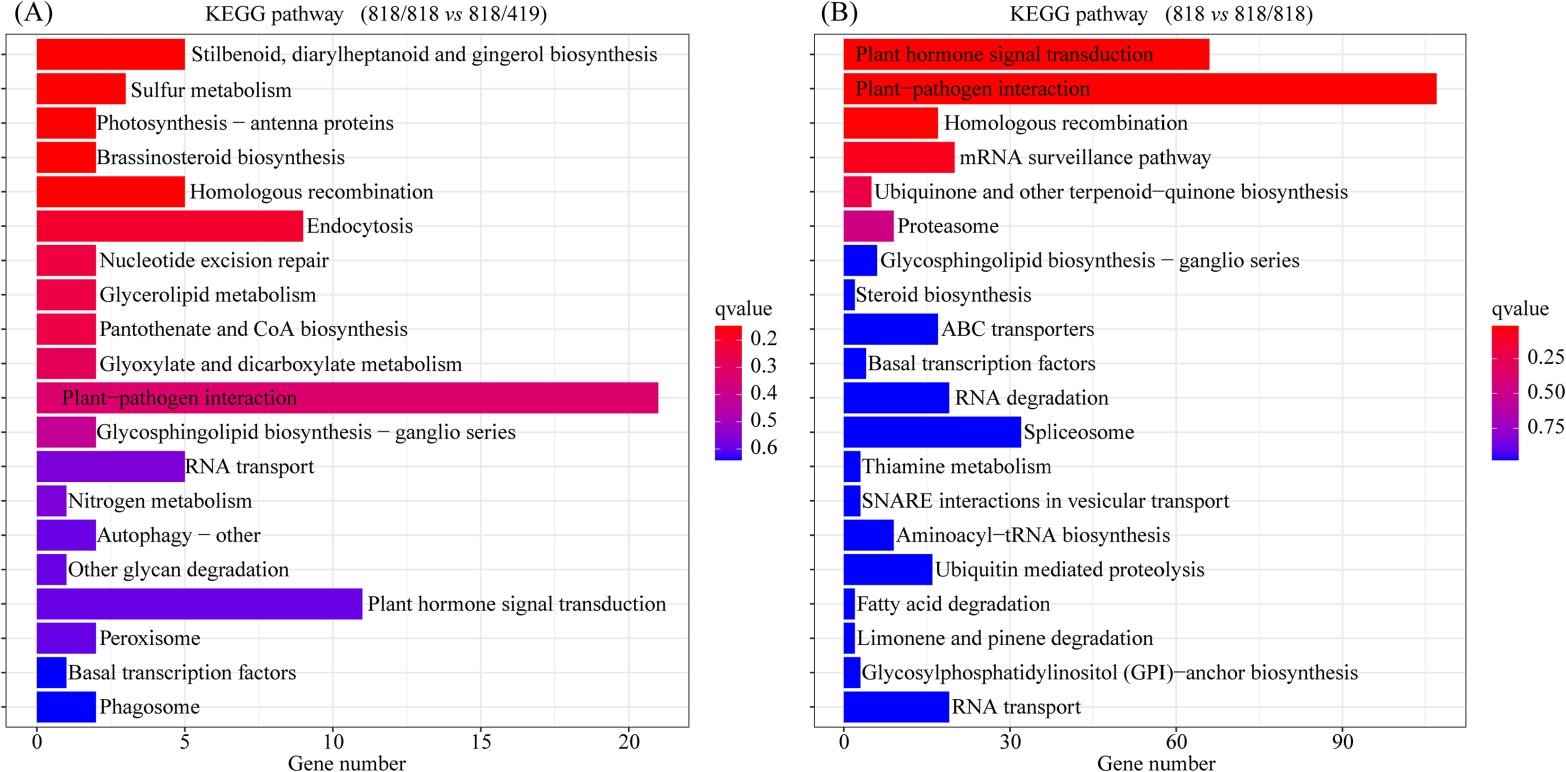
KEGG enrichment of miRNA target genes in different tea grafting combinations. **(A)** Comparison between ungrafted ‘818’ and self-grafted ‘818/818’; **(B)** Comparison between self-grafted ‘818/818’ and hetero-grafted ‘818/419’. Color represents adjusted q-value.

Small RNA sequencing of scions from ‘818’, ‘818/818’, and ‘818/419’ yielded 109.68 million clean reads, with Q30 > 85%, and sequence lengths predominantly 21–24 nt, characteristic of plant miRNAs. A total of 615 miRNAs were identified (88 known, 527 novel). Differential expression analysis revealed 126 differentially expressed miRNA (DEMs;48 upregulated, 78 downregulated) in ‘818 *vs* 818/818’ and 31 DEMs (15 upregulated, 16 downregulated) in ‘818/818 *vs* 818/419’ (**Supplementary Figure S1B**). KEGG enrichment of DEM targets indicated involvement in plant hormone signaling, pathogen interaction, homologous recombination, and mRNA surveillance in ‘818 *vs* 818/818’ (**Figure 4A**); and additionally sulfur metabolism, steroid biosynthesis, diarylheptanoid/gingerol biosynthesis, endocytosis, and photosynthesis regulation in ‘818/818 *vs* 818/419’ (**Figure 4B**). These data suggest that grafting mediates physiological adaptation and defense enhancement via miRNA-regulated networks governing defense, tissue repair, and metabolism.

### 2.5 Metabolomic reprogramming associated with enhanced resistance

**Figure 5:**
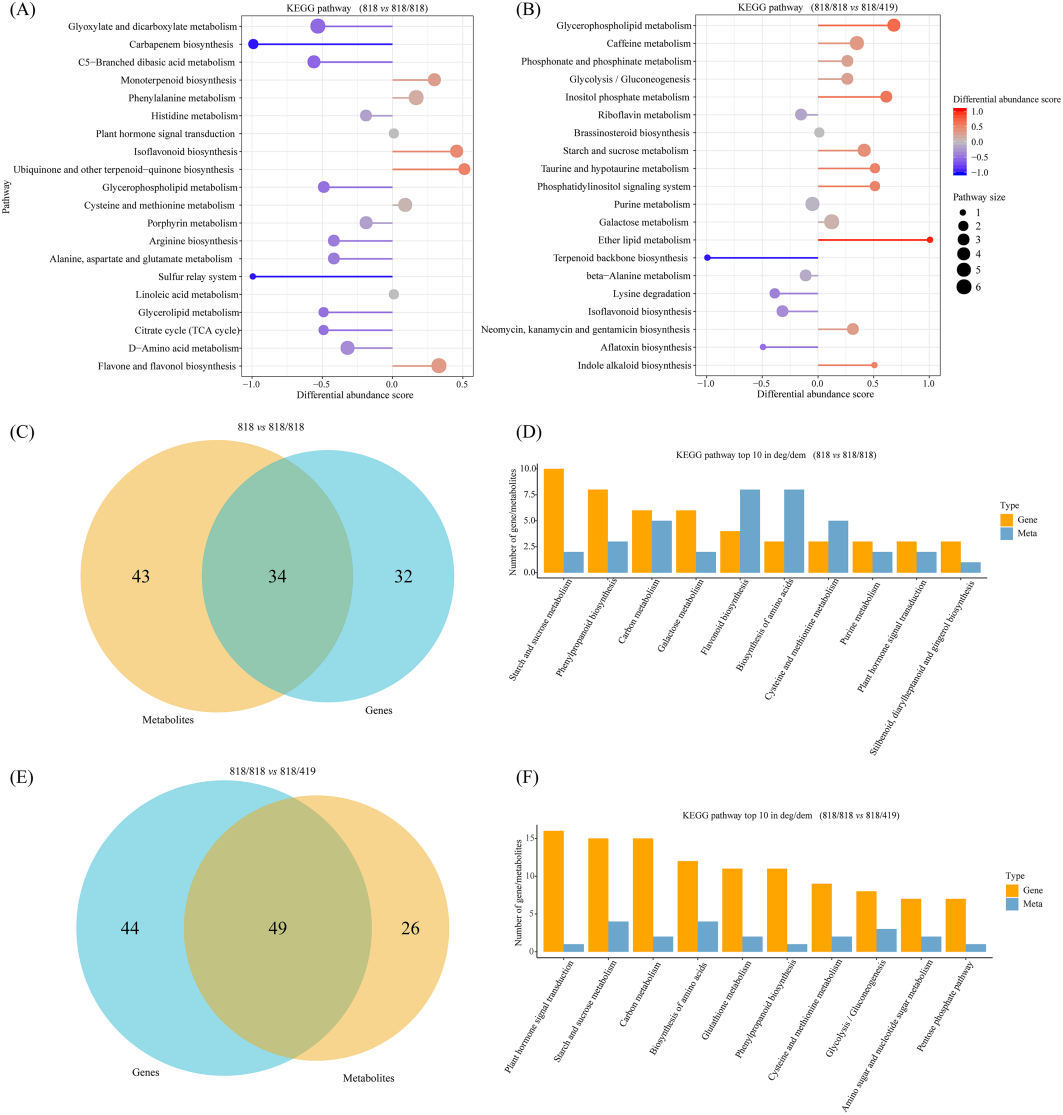
Integrated KEGG pathway enrichment analysis of differential metabolites and co-expressed genes in different tea grafting combinations. **(A, B)** Visualization of KEGG pathway enrichment based on differential metabolites in ‘818 *vs* 818/818’and ‘818/818 *vs* 818/419’. For each pathway, the horizontal position of the dot represents the Differential Abundance (DA) Score (ranging from -1 to 1), indicating the overall up- or down-regulation trend of all annotated metabolites within that pathway. The distance from the center line reflects the absolute value of the DA Score. Dot size corresponds to the number of differential metabolites mapped to the pathway. Dot and line color reflects the statistical significance (p-value), with a gradient from blue (less significant) to red (more significant); **(C, E)** Venn diagrams illustrating overlapping KEGG-enriched pathways between ‘818 *vs* 818/818’ and ‘818/818 *vs* 818/419’ for differential metabolites and co-expressed differential genes and metabolites; **(D, F)** Top 10 KEGG-enriched pathways for co-expressed differential genes and metabolites in ‘818 *vs* 818/818’and ‘818/818 *vs* 818/419’. Bar height indicates pathway activity level within the measured samples.

Widely targeted metabolomics analysis detected 1870 metabolites in scion leaves. Differential analysis identified 430 differential metabolites (261 upregulated, 169 downregulated) in ‘818 vs 818/818’, and 361 (171 upregulated, 190 downregulated) in ‘818/818 *vs* 818/419’ (**Supplementary Figure S1C**). KEGG enrichment showed upregulated metabolites in ‘818 *vs* 818/818’ enriched in monoterpenoid biosynthesis, phenylalanine metabolism, and ubiquinone/terpenoid-quinone biosynthesis; downregulated ones were enriched in carboxylic acid metabolism, the glyoxylate cycle, glycerophospholipid metabolism, and beta-alanine metabolism (**Figure 5A**). In the ‘818/818 *vs* 818/419’ comparison, upregulated metabolites were enriched in glycerophospholipid metabolism, caffeine metabolism, and phosphatidylinositol signaling; downregulated metabolites were involved in acetyl-CoA and lipid pathways (**Figure 5B**). These results indicate that grafting modulated primary energy metabolism and enhanced accumulation of secondary metabolites related to quality and disease resistance. Resistant rootstock ‘419’ further amplified disease-relevant metabolic pathways, promoting biosynthesis and accumulation of defense compounds that improve resistance and quality.

### 2.6 Integrated multi-omics reveals coordinated defense networks

Integrative analysis of transcriptomic and metabolomic data revealed that co-expressed DEGs and metabolites in the comparison ‘818 *vs* 818/818’ were enriched in 34 KEGG pathways, predominantly starch and sucrose metabolism, phenylalanine metabolism, carbon metabolism, and isoflavonoid biosynthesis (**Figures 5C, D**). In ‘818/818 *vs* 818/419’, 49 enriched pathways were identified, including plant hormone signal transduction, carbon metabolism, glutathione metabolism, amino acid biosynthesis, and photosynthesis regulation (**Figures 5E, F**). These results suggest that grafting modulates basal metabolism and secondary metabolite synthesis, while resistant rootstocks activate comprehensive defense-related gene expression and metabolite accumulation, enhancing the plant’s immune response. Complementary miRNA sequencing data corroborated the observed enrichment of target genes in hormone signaling and glutathione metabolism pathways, underscoring the miRNA-mediated post-transcriptional regulation coordinating these responses.

### 2.7 qRT-PCR validation of miRNA-mRNA pairs

**Figure 6:**
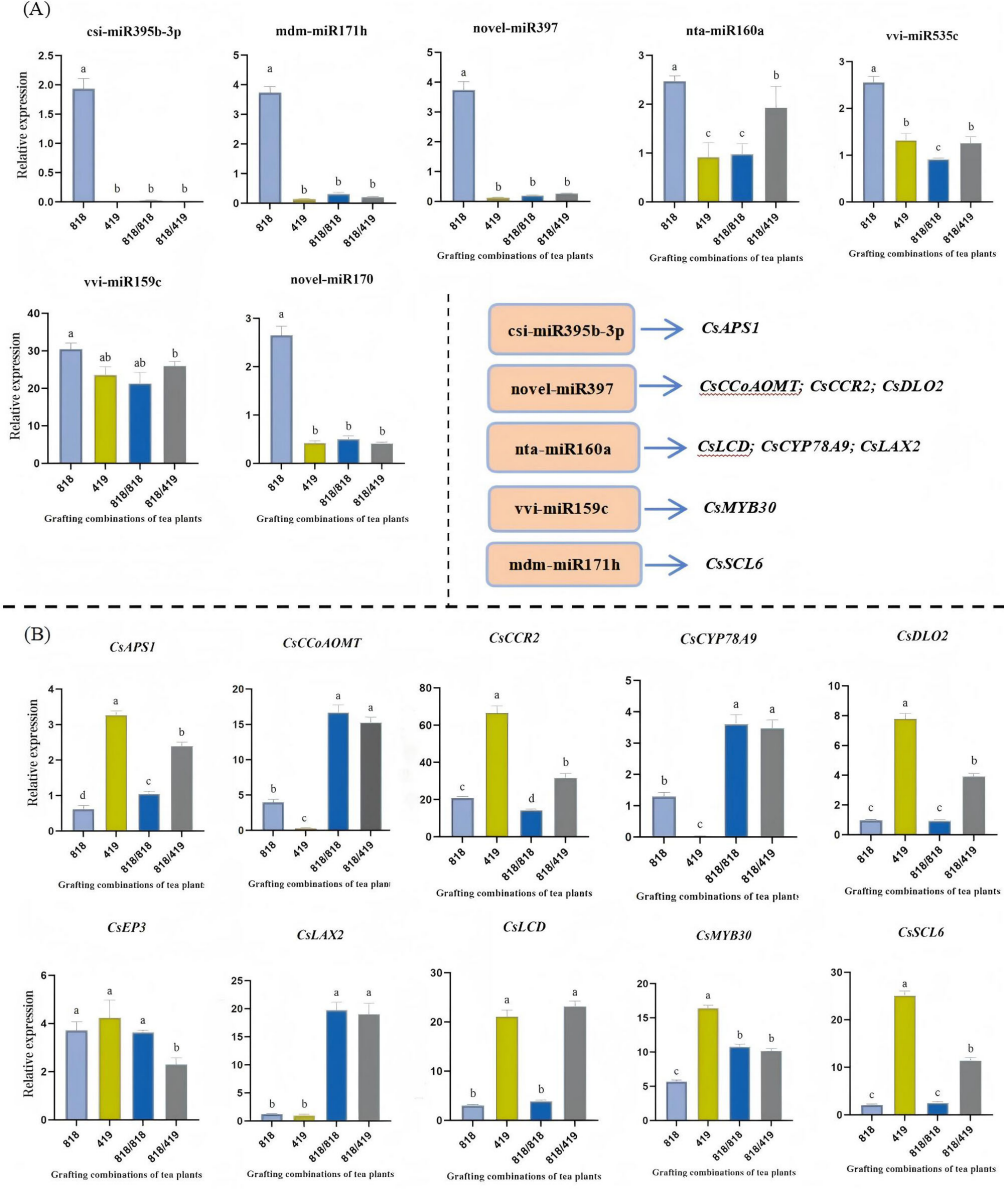
Experimental validation of key omics findings by qRT-PCR. **(A)** Relative expression levels of selected differentially expressed miRNAs; **(B)** Relative expression levels of selected differentially expressed genes. Expression levels were validated in the relevant tea grafting combinations. Different letters above the bars indicate significantly different lesion diameters according to Duncan’s multiple comparison test (*p* < 0.05).

To verify the sequencing data, we selected seven differentially expressed miRNAs and ten associated target genes for qRT-PCR analysis. The expression patterns were largely consistent with the high-throughput sequencing results, revealing predominant inverse correlations between the miRNAs and their putative targets (**Figure 6**; **Supplementary Tables S2, S3**). All seven validated miRNAs showed significant downregulation in the resistant hetero-graft ‘818/419’ compared to the susceptible ungrafted ‘818’ (*p* < 0.05). Among them, csi-miR395b-3p, novel-miR397, nta-miR160a, mdm-miR171h, novel-miR170, and vvi-miR535c were also downregulated in self-grafted ‘818/818’ and the resistant rootstock ‘419’, whereas vvi-miR159c was specifically suppressed in ‘818/419’.

Conversely, the expression of most target genes was significantly upregulated. For example, downregulation of csi-miR395b-3p was associated with strong induction of its target involved in sulfur assimilation, *CsAPS1*, in both ‘419’ and ‘818/419’. Suppression of novel-miR397 correlated with marked upregulation of three lignin biosynthesis-related genes—*CsCCoAOMT*, *CsCCR2*, and *CsDLO2*—in the resistant and hetero-grafted plants. Downregulation of nta-miR160a coincided with elevated levels of *CsLAX2* (an auxin transporter) and *CsCYP78A9*, indicating enhanced auxin signaling. Similarly, downregulation of vvi-miR159c and mdm-miR171h aligned with upregulation of their respective putative targets: the transcription factor *CsMYB30* and the signaling gene *CsSCL6*.

Notably, the self-grafted ‘818/818’ typically displayed intermediate expression changes, whereas hetero-grafting with ‘419’ elicited the most substantial transcriptional reprogramming. These validated miRNA-mRNA pairs constitute a core post-transcriptional regulatory network that underlies the grafting-enhanced defense response.

## 3 Discussion

Grafting technology, as an effective strategy to enhance plant resistance, has shown substantial application value across various crops and economic plants (Goldschmidt, 2014; Kyriacou et al., 2017). In this study, we elucidated how the resistant rootstock ‘Qianmei 419’ (‘419’) improves resistance to *C. camelliae* in the susceptible tea scion ‘Qianmei 818’ (‘818’) by integrating transcriptomic, metabolomic, and small RNA omics analyses. Our study showed that disease severity (assessed by the average lesion diameter of inoculated leaves) was 98.71% lower in hetero-grafted ‘818/419’ plants than is ungrafted ‘818’. However, the complex molecular mechanisms underlying this grafting-conferred resistance required further clarification. By providing combined physiological, biochemical, and multi-omics data, this work reveals a coordinated network involving hormone signaling, miRNA regulation, and metabolic reprogramming during grafting, thereby providing a theoretical framework and practical insights for breeding disease-resistant tea cultivars.

### 3.1 Hormonal and enzymatic bases of graft enhanced resistance to *C. camelliae*

Grafting markedly altered the scion’s hormonal profile, especially in the heterograft ‘818/419’, where IAA and GA_3_ contents increased significantly. These growth-promoting hormones likely support wound healing and vigor, thereby contributing to enhanced resistance (Sharma and Zheng, 2019; Zhang et al., 2022). In parallel, MeSA, a mobile derivative of SA associated with defense priming (Lan et al., 2025), also accumulated strongly in both self- and hetero-grafted plants, suggesting that grafting reprograms SA metabolism toward enhanced defensive readiness. At the enzymatic level, heterografting with the resistant rootstock elevated PAL activity, a key enzyme for lignin and flavonoid biosynthesis (Dixon et al., 2002), while reducing SOD and POD activities, indicating a shift from broad antioxidant responses toward specialized defense pathways. Taken together, these findings suggest that grafting, particularly onto resistant rootstocks, enhances tea plant resistance to *C. camelliae* by promoting IAA- and GA3-mediated growth, strengthening SA-derived MeSA defense signaling, and activating PAL-driven secondary metabolism.

### 3.2 Resistant rootstocks mediate *C. camelliae* resistance through miRNA-regulated gene expression

The miRNA-mRNA expression patterns described above provide a mechanistic basis for understanding how resistant rootstocks influence scion defense responses. Building on these results, small RNA sequencing and qPCR validation further revealed that grafting, particularly hetero-grafting with resistant rootstock ‘419’, induces pronounced shifts in scion miRNA expression profiles, accompanied by reciprocal changes in their target or putative target genes. For example, csi-miR395b-3p was significantly downregulated in ‘419’ and ‘818/419’, while its target *CsAPS1* and related sulfur metabolism gene *CsLCD* were upregulated, a result consistent with the known role of miR395’s in sulfur homeostasis (Kawashima et al., 2009; Tao et al., 2012). Enhanced sulfur metabolism is known to improve antioxidant capacity via glutathione synthesis and production of sulfur-containing defense compounds (Bednarek et al., 2009; Hacham et al., 2025).

Our study resulted in the identification of a novel miRNA, that we propose to name ‘novel-miR397’ (**Supplementary Table S4**). The downregulation of this novel-miR397 correlated with increased expression of three genes, *CsCCoAOMT*, *CsCCR2*, and *CsDLO2*, that are known to be linked with reinforcing lignin biosynthesis and structural barriers (Huang et al., 2021; Xue et al., 2019; Wang et al., 2019). Reduced nta-miR160a was linked to elevated *CsLAX2* and *CsCYP78A9*, indicating enhanced auxin transport and organ development (Hao et al., 2022; Moreno-Piovano et al., 2017; Sotelo-Silveira et al., 2013). Downregulation of vvi-miR159c corresponded with higher expression of *CsMYB30*, a gene known to code for a stress-responsive transcription factor (Millar et al., 2019; Fichman et al., 2020), while reduced mdm-miR171h matched increased expression of *CsSCL6*, a gene involved in hormone signaling (Wang et al., 2020b; Yang et al., 2010). These coordinated miRNA–mRNA shifts, with intermediate changes in self-grafted ‘818/818’, support a systemic, miRNA-driven regulatory mechanism in graft-mediated resistance, a finding consistent with the role proposed by Melnyk et al. (2011b) for long-distance miRNA transport.

### 3.3 Graft-induced transcriptional and metabolic reprogramming enhances resistance while preserving scion quality traits

Integrated metabolomic and transcriptomic analyses demonstrated that grafting, especially hetero-grafting with resistant rootstock ‘419’, reprograms scion metabolism and gene expression. In ‘818/419’, upregulated metabolites were enriched in glycerophospholipid metabolism, phosphatidylinositol signaling, and caffeine biosynthesis, processes linked in previous studies to membrane stability, immune signal transduction, and pathogen inhibition (Guo et al., 2023; Abd-El-Haliem and Joosten, 2017; Zhang et al., 2010). Integrated metabolomic and transcriptomic analyses revealed that in ‘818/419’ scion leaves, differential metabolites and genes in pathways such as plant hormone signal transduction, glutathione metabolism, and phenylpropanoid biosynthesis exhibited synergistic expression, demonstrating a tight transcriptional-metabolic coupling. This further demonstrates that the increased expression of key genes such as *CsAPS1*, *CsCCoAOMT*, *CsCCR2*, *CsDLO2*, *CsLAX2* and *CsLCD* promotes plant hormone and lignin synthesis, as well as signal transduction. These changes parallel the downregulation of their regulatory miRNAs, reinforcing the link between post-transcriptional control and metabolic adaptation.

Notably, the defense-related metabolic reprogramming induced by the resistant rootstock did not compromise the key quality-determining metabolites of the scion. ‘818/419’ was shown in our study to hold important traits associated with tea quality. It retained the characteristic high EGCG content of scion ‘818’, while showing increased EC, EGC, and L-theanine. Furthermore, it preserved two desirable traits of rootstock ‘419’: high ECG and caffeine contents. These findings demonstrate that grafting onto a resistant rootstock triggers extensive miRNA-directed regulatory cascades, broad transcriptional reprogramming, and differential enrichment of defense-associated pathways, thereby conferring robust phylloxera resistance while maintaining the scion’ s canonical levels of key quality-associated metabolites and permitting the accumulation of additional compounds contributed by the rootstock.

### 3.4 An integrated model of grafting-enhanced resistance to *C. camelliae*

**Figure 7:**
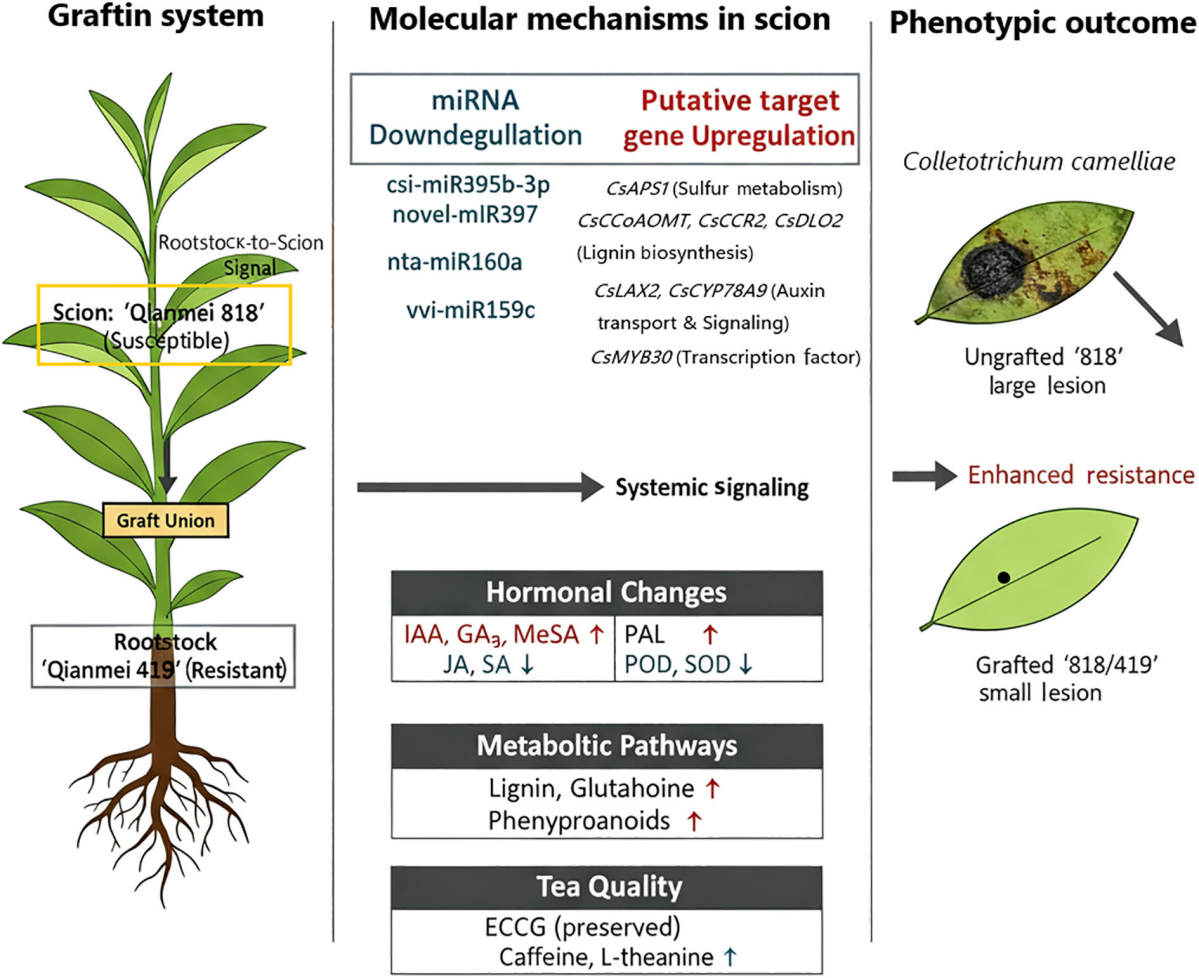
Multi-omics model of grafting enhanced resistance to *C. camelliae* in tea plants. Grafting the susceptible scion ‘Qianmei 818’ onto the resistant rootstock ‘Qianmei 419’ triggers systemic signaling that reprograms the scion. (1) Key miRNAs shift (e.g., downregulation of csi-miR395b-3p and novel-miR397); (2) Putative target genes are upregulated, activating defense pathways including sulfur metabolism (*CsAPS1*, *CsLCD*), lignin biosynthesis (*CsCCoAOMT*, *CsCCR2*, *CsDLO2*), and hormone signaling/transport (*CsLAX2*, *CsCYP78A9*); (3) Phytohormone homeostasis shifts, with increased IAA, GA3, and MeSA, and decreased JA and SA; (4) Defense enzyme activities are modulated (higher PAL; lower POD and SOD); (5) The metabolome is remodeled, enriching glycerophospholipid metabolism and phenylpropanoid biosynthesis while preserving or enhancing quality-related metabolites (EGCG, caffeine, L-theanine). Together, these changes strengthen structural barriers (lignin), augment chemical defenses, and prime immunity, resulting in markedly reduced lesion development following *C. camelliae* infection.

This study integrates multi-omics and phenotypic data to propose a hierarchical, systemic resistance network orchestrated by grafting-responsive miRNAs. The enhanced resistance in ‘818/419’ scions is a coherent cascade initiated by rootstock-derived signals (**Figure 7**). Specifically, the differential expression of key miRNAs (e.g., csi-miR395b-3p, novel-miR397) in the scion fine-tunes the transcriptome, leading to the upregulation of target genes governing sulfur assimilation (*CsAPS1*), lignin biosynthesis (*CsCCoAOMT*, *CsCCR2*), and auxin transport (*CsLAX2*). This transcriptional reprogramming subsequently drives a metabolic shift toward defense-ready states, characterized by the accumulation of MeSA, IAA for signaling, enhanced lignin deposition for structural reinforcement, and elevated glutathione for redox homeostasis. Consequently, the tea plant’s strategy pivots from a broad antioxidant response (reflected in altered SOD/POD activities) to a targeted deployment of specialized physical and biochemical barriers, which directly translates into the dramatic 98.71% reduction in lesion diameter.

Grafting-induced resistance conferred by resistant rootstocks is well-documented in various crops, including grapevine (Cookson et al., 2013), apple (Jensen et al., 2012), watermelon (Zhang et al., 2025), and citrus (Wang et al., 2025). The enhanced resistance in these grafted systems is primarily attributed to long-distance signaling mechanisms. In grafting systems, mobile signals such as miRNAs, hormones, and metabolites are likely transported from rootstock to scion through the phloem via source-sink dynamics, thereby reprogramming scion responses. In this study, we postulate that similar long-distance signaling enhances scion defense against *C. camelliae* in grafted tea plants. Numerous studies have established that 21–24 nt small RNAs, including many of the miRNAs identified here, can function as mobile silencing signals that traverse graft junctions in a selective, Argonaute-dependent manner (Melnyk et al., 2011a; Li et al., 2021). Once in the recipient scion tissue, these mobile miRNAs-transported as single-or double-stranded forms bound to Argonaute proteins-trigger target mRNA cleavage, translational repression, or epigenetic modifications such as DNA methylation. This process is frequently amplified by scion-localized RNA-dependent RNA polymerases and mirrors endogenous long-distance signaling events, such as the well-characterized shoot-to-root movement of miR399 during phosphate homeostasis (Lin et al., 2008; Pant et al., 2008). The observed changes in miRNA expression levels in the scions of grafted tea plants may therefore be associated with the mobility of miRNAs between rootstock and scion. Additionally, hormones such as MeSA, which accumulated in our hetero-grafts, represent another class of mobile signals, as volatile or phloem-transported SA derivatives, they prime systemic acquired resistance across distances (Park et al., 2007). Similarly, defense-related metabolites (e.g., from phenylpropanoid and glutathione pathways) may be conveyed with photoassimilates via the phloem, thereby enhancing scion defense compounds. Currently, our data provide indirect evidence for the long-distance transport of these substances in grafted tea plants. Future studies employing tracer assays or direct phloem profiling are needed to validate these transport mechanisms. Notably, while our multi-omics approach provides robust correlative support for miRNA-mRNA networks, functional validations such as miRNA or gene overexpression and silencing are crucial to establish causality and delineate the mechanistic details of grafting-conferred immunity.

## 4 Conclusion

Through an integrative multi-omics approach, this study delineates the molecular mechanisms underlying grafting-enhanced *C. camelliae* resistance in tea plants. The resistant rootstock ‘419’ remodels hormonal homeostasis, modulates key miRNAs and their targets, and reprograms metabolic pathways, thereby significantly improving disease resistance and simultaneously enhancing tea quality in the susceptible scion ‘818’. These insights provide a theoretical foundation for disease-resistant breeding in tea and support the advancement of sustainable, environmentally friendly disease control strategies. Future work will focus on elucidating the mechanisms governing long-distance miRNA transport between rootstock and scion and evaluating grafted tea plant resistance under complex field conditions to optimize practical applications of grafting technology in tea cultivation.

## 5 Materials and methods

### 5.1 Plant materials and grafting scheme

Two-year-old tea plants (*Camellia sinensis*) cultivated by the Guizhou Provincial Tea Research Institute were used in this study. The *C. camelliae* susceptible cultivar ‘Qianmei 818’ (hereafter ‘818’) served as the scion, while the *C. camelliae* resistant cultivar ‘Qianmei 419’ (hereafter ‘419’) was employed as the rootstock for grafting. Grafting was conducted in spring using the cleft grafting method described by Liu et al. (2017). Two graft combinations were established: self-grafted (‘818/818’; i.e., ‘818’ scion onto ‘818’ rootstock) and hetero-grafted (‘818/419’; i.e., ‘818’ scion onto ‘419’ rootstock). Following grafting, seedlings underwent standard field management and were cultivated for one year to ensure stable growth prior to downstream experiments. This one-year period was chosen to ensure complete graft union formation, stable systemic signal transmission between rootstock and scion, and consistent physiological maturity prior to inoculation experiments, as preliminary field trials indicated that shorter periods resulted in incomplete healing and variable resistance phenotypes.

### 5.2 Detached leaf inoculation assay for *C. camelliae*

The resistance of grafted tea plants to *C. camelliae* was assessed using the detached leaf inoculation assay of Yoshida and Takeda (2006). The first fully expanded leaves (third leaf from the apex) were excised from healthy plants belonging to four groups: ungrafted ‘818’, ungrafted ‘419’, self-grafted ‘818/818’, and hetero-grafted ‘818/419’. The leaves were surface-sterilized by wiping with 75% ethanol and rinsed with sterile distilled water. They were blotted dry, then placed in 90-mm-diameter Petri dishes on water-soaked sterile filter paper, with their adaxial side up. The leaves were then immediately inoculated with *C. camelliae* stain GC-2. This strain was originally isolated from anthracnose-infected tea leaves in Guizhou Province, morphologically and phylogenetically identified as *C. camelliae* based on multi-gene (*ITS*, *GAPDH*, *ACT*, *CHS*) analysis combined with pathogenicity tests (as detailed in Chen, 2023, Master’s thesis, Guizhou University), and preserved in the Plant Protection Laboratory, Guizhou institute of tea science. Long-term stock cultures are maintained in 20% glycerol at -80°C, with working cultures stored on potato dextrose agar (PDA) slants at 4°C. Mycelial plugs, 5 mm in diameter, were excised from the growing margin of a 7-day old colony incubated on potato dextrose agar (PDA) at 25°C in the dark. On each leaf, a mycelial plug was deposited on the left side of the midrib, with the mycelium in direct contact with the plant tissue. A plug of non-inoculated PDA was placed on the right side of the midrib as a control. The Petri dishes were then covered with moist cotton, and incubated at 28°C in darkness. The moisture of the cotton was monitored during incubation and water was added as needed to maintain saturated humidity around the leaves. Disease development was assessed by measuring the diameter of the lesions 3 days after inoculation. To facilitate the observation of lesions with low development (diameters smaller than 5 mm) the mycelial plugs were removed from the leaves prior to their examination. Each treatment included 10 replicate leaves, and the whole experiment was carried out three times independently.

### 5.3 Physiological parameter measurement

Healthy shoots comprising one bud and two leaves were excised from plants with consistent growth status in all four treatment groups (‘818’, ‘419’, ‘818/818’, and ‘818/419’). The shoots were immediately flash-frozen in liquid nitrogen and stored at -80°C until analyses were carried out. The activity of defense-related enzymes peroxidase (POD), superoxide dismutase (SOD) and phenylalanine ammonia-lyase (PAL) was quantified using commercial colorimetric assay kits (Suzhou Kaiming, China) following the manufacturer’s instructions.

The levels of plant hormones including indole-3-acetic acid (IAA), gibberellic acid (GA_3_), salicylic acid (SA), and jasmonic acid (JA), alongside major catechins (epigallocatechin gallate [EGCG], epicatechin [EC], epigallocatechin [EGC], and epicatechin gallate [ECG]), L-theanine and caffeine, were determined via high-performance liquid chromatography (HPLC). Metabolites were extracted using methanol, and quantification was performed based on calibration curves generated from purified standards, with relative standard deviations maintained below 15%. Concentrations (µg/g) were calculated as follows:

Concentration = (Instrument Reading x Final Extract Volume [mL] x Dilution Factor)/Sample Mass [g].

For each of the four plant treatment groups, we analyzed three biological replicate samples, each sample being composed of shoots from five plants.

### 5.4 Multi-omics data analysis

#### 5.4.1 Sample collection, RNA, and metabolite extraction

Samples of shoots comprising one bud and the two first leaves were collected from plants of each of three plant groups (‘818’ ungrafted, ‘818/818’ self-grafted, and ‘818/419’ hetero-grafted), immediately flash-frozen in liquid nitrogen, and stored at -80°C. Total RNA extraction was performed using a Plant Total RNA Extraction Kit (Tiangen Biotech Co., Ltd., Beijing, China), following the manufacturer’s protocol. RNA quality and concentration were evaluated using a NanoDrop 2000 spectrophotometer and Agilent 2100 Bioanalyzer. Only high-quality RNA samples were subjected to subsequent mRNA and micro RNA (miRNA) sequencing. Metabolites were extracted using a methanol: acetonitrile: water solvent mixture (1:2:1, v/v/v). Ultimately, each group was represented by three biological replicates, each containing pooled material from five plants.

#### 5.4.2 RNA-Seq and sRNA-Seq analysis

Separate mRNA and sRNA libraries were constructed and sequenced on the Illumina platform. Clean mRNA reads were aligned to the tea plant reference genome Shuchazao V2 (Xia et al., 2020) with HISAT2 v2.0.4 (-{{-}}-dta -p 6 -{{-}}-max-intronlen 5000000), and transcripts were assembled using StringTie v2.2.1. Differentially expressed genes (DEGs) were identified by DESeq2 (|log_2_FoldChange| > 1.5, *p* < 0.05, Benjamini-Hochberg FDR). Functional annotation was performed against Gene Ontology (GO) and Kyoto Encyclopedia of Genes and Genomes (KEGG) databases.

For small RNA data, reads were mapped with Bowtie (-v 0 -S -f) for known miRNAs and miRDeep2 (-g 50000 -l 250 -d -m 10 -v -P -n d) for novel miRNAs. Differentially expressed miRNAs (DEMs) were identified using DESeq2 (|log_2_FoldChange| > 0.58, *p* < 0.05, FDR). Target genes of DEMs were predicted using TargetFinder v1.6, and annotated via BLAST searches against public databases, followed by KEGG pathway enrichment analyses.

#### 5.4.3 Widely targeted metabolomics analysis

Metabolite profiling was conducted using a widely targeted metabolomics approach on an Acquity I-Class PLUS ultra-performance liquid chromatography (UPLC) system coupled with an AB Sciex QTRAP 6500+ mass spectrometer. Metabolite identification and quantification were performed against the Biomarker Technologies proprietary database GB-PLANT supplemented by public databases (KEGG Compound, HMDB, and Lipidmaps). Raw data were processed using Analyst v1.7.2 for peak integration, calibration, and normalization. Differentially accumulated metabolites (DAMs) were identified via OPLS-DA with thresholds of Variable Importance in Projection (VIP) scores ≥ 1,fold change>1 and *p* < 0.05. Metabolites were subsequently annotated and mapped to metabolic pathways using the KEGG database. Integrated multi-omics analyses combining widely targeted metabolomics with transcriptomic and small RNA datasets were performed to elucidate the molecular regulatory networks underpinning grafting-induced anthracnose resistance.

### 5.5 qRT-PCR validation

To validate RNA-Seq and sRNA-Seq findings, representative differentially expressed miRNAs and their target mRNAs were selected for quantitative real-time PCR (qRT-PCR). miRNA cDNA synthesis employed stem-loop primers, whereas mRNA was reverse transcribed via a universal reverse transcription kit (TAKARA Bio Inc., Japan). qRT-PCR was performed using SYBR Green on an ABI 7500 Real-Time PCR System. Pc-222-3p and *CsGAPDH* served as endogenous controls for miRNA and mRNA quantification, respectively. Relative expression was calculated using the 2^–ΔΔCt^ method. Each reaction included three biological replicates and three technical replicates. Primer sequences are listed in **Supplementary Table S1**.

### 5.6 Statistical analysis and data integration

Data were analyzed by one-way analysis of variance (ANOVA) using SPSS 22.0. Significant differences between groups were determined by Duncan’s multiple range test at a significance threshold of *p* < 0.05.

## Data Availability

The datasets presented in this study can be found in online repositories. The names of the repository/repositories and accession number(s) can be found in the article/**Supplementary Material**.
