# Supplementary material for: Multi-omics analysis of molecular mechanisms driving the grafting- enhanced resistance of tea plants to Colletotrichum camelliae
Source: Front Plant Sci. 2026 Jan 14;16:1750493. doi: 10.3389/fpls.2025.1750493 (PMC12847328; doi:10.3389/fpls.2025.1750493)
Supplement: Supplementary file 1 [file Table1.docx]

Supplementary Material

# Supplementary Figures and Tables

For more information on Supplementary Material and for details on the different file types accepted, please see [here](https://www.frontiersin.org/guidelines/author-guidelines#supplementary-material).

## Supplementary Figures


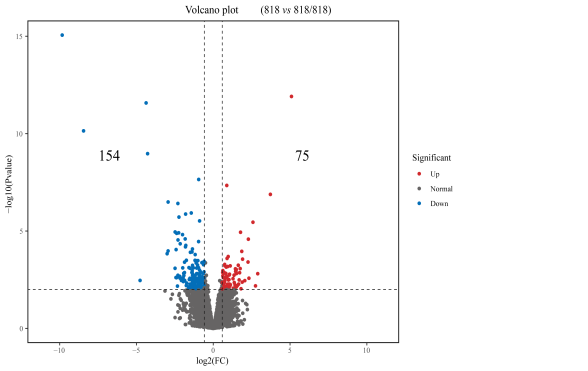

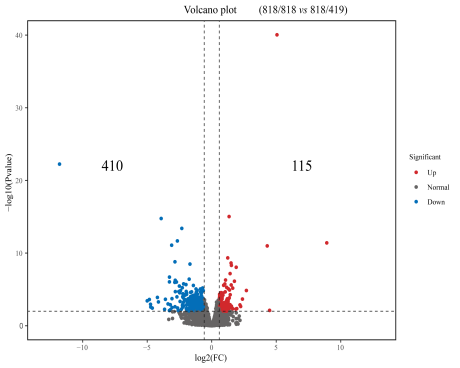

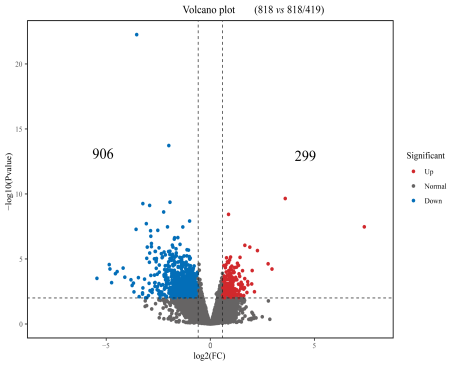

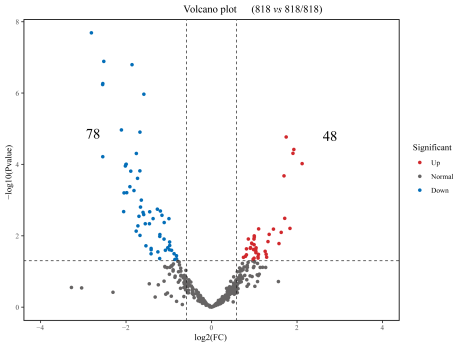

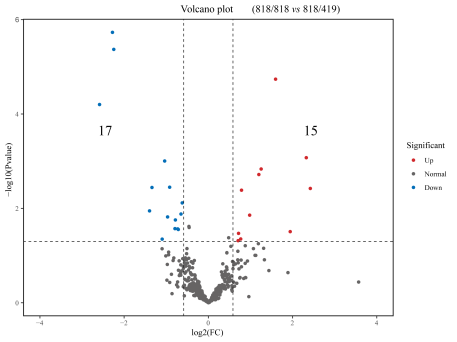

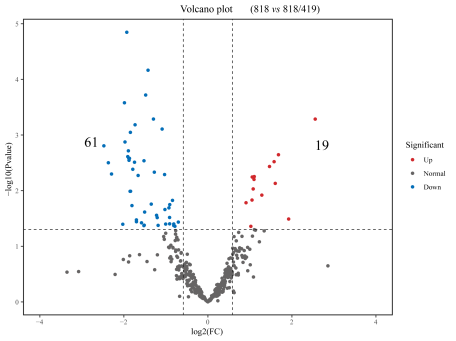

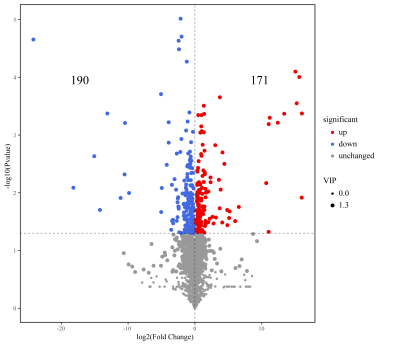

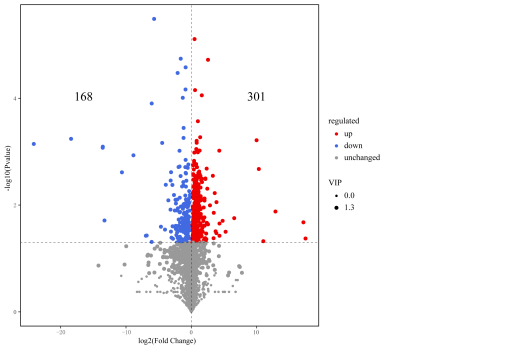


(818 *vs* 818/818)

(818/818 *vs* 818/419)

(818 *vs* 818/419)


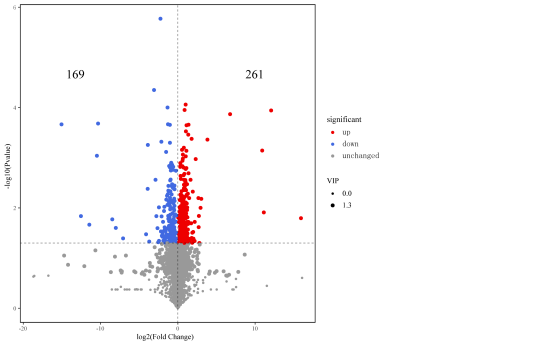


(B)

(C)

(A)

Figure S1. Volcano plots of differentially expressed genes, miRNAs, and metabolites among different tea grafting systems.

(A) Differentially expressed genes between different tea grafting systems; (B) Differentially expressed miRNAs between different tea grafting systems; (C) Differentially expressed metabolites between different tea grafting systems.

Red dots indicate significantly upregulated features, blue dots indicate significantly downregulated features, and gray dots indicate no significant change.

Selection criteria are as follows:

Differentially expressed genes (DEGs): |log₂FoldChange| ≥ 1.5, *p* < 0.01;

Differentially expressed miRNAs (DEMs): |log₂FoldChange| > 0.58, *p* < 0.05;

Differentially expressed metabolites (DEMs): VIP ≥ 1 and *p* < 0.05.

## Supplementary Tables

Table S1 qPCR Primers for mRNA and miRNA

| **Primer Name** | Primer Sequence (5'to3') |
| --- | --- |
| *CsGAPDH*-F | CACGGTCAATGGAAGCATCAT |
| *CsGAPDH*-R | GCAGCAGCCTTATCCTTATCAG |
| *CsAPS1*-F | TGGCGTTCTTTGATCCCTTGA |
| *CsAPS1*-R | CCAACCACCGGGACACATAA |
| *CsMYB30*-F | TAGCCCTAGTGAGGGGACTG |
| *CsMYB30*-R | TGGTTGTTGTCTCATCCACTG |
| *CsDLO2*-F | GAGTGTGGAGCATAGGGTGAC |
| *CsDLO2*-R | TCAGCTCCTCTAAAGGCTCC |
| *CsEP3*-F | GACCGCCTTGTGGTATTGGAT |
| *CsEP3*-R | TGTGTTTCCACCATCGCATTC |
| *CsCYP78A9*-F | GTGGACGCTGAGATGGAGTT |
| *CsCYP78A9*-R | TCAAACTCGTGCAAAAGCGAG |
| *CsCCR2*-F | ACTCAGGAAAAACTACCCGAACT |
| *CsCCR2*-R | TCCAACCCAGCTTCTGCAAT |
| *CsLCD*-F | CCAGTATGGCAATGGTTGGC |
| *CsLCD*-R | TTGGTGCCTGATGGTAGTGT |
| *CsCCoAOMT*-F | TGTTGAGCGATGGTAAGTTGAAA |
| *CsCCoAOMT*-R | CACCAGTTTTATCGCTTGCTCG |
| *CsSCL6*-F | TTCGCACAGCACCTCTTTCA |
| *CsSCL6*-R | TGCTTTCAATTCTGGGCTGG |
| *CsLAX2*-F | TTTGTGGTTGTGTGGGTCCTT |
| *CsLAX2*-R | TTGAGGAGGAGGGCATTGGT |
| Pc-222-3p-RT | GTCGTATCGACTGCAGGGTCCGAGGTATTCGCAGTCGATACGACTCGGCA |
| Pc-222-3p-F | CTTTCCAAGACCACCCATGC |
| csi-miR395b-3p-RT | GTCGTATCGACTGCAGGGTCCGAGGTATTCGCAGTCGATACGACGAGTTC |
| csi-miR395b-3p-F | CGCTGAAGTGTTTGGGGGAA |
| novel_miR170-RT | GTCGTATCGACTGCAGGGTCCGAGGTATTCGCAGTCGATACGACCCAATT |
| novel_miR170-F | GGCTTGCATACGCACCTGAAT |
| vvi-miR535c-RT | GTCGTATCGACTGCAGGGTCCGAGGTATTCGCAGTCGATACGACGCGTGC |
| vvi-miR535c-F | CGCTGACAACGAGAGAGAGCA |
| vvi-miR159c-RT | GTCGTATCGACTGCAGGGTCCGAGGTATTCGCAGTCGATACGACTAGAGC |
| vvi-miR159c-F | CGCTTTGGATTGAAGGGAGCT |
| nta-miR160a-RT | GTCGTATCGACTGCAGGGTCCGAGGTATTCGCAGTCGATACGACTGGCAT |
| nta-miR160a-F | CGTGCCTGGCTCCCTGTATG |
| mdm-miR171h-RT | GTCGTATCGACTGCAGGGTCCGAGGTATTCGCAGTCGATACGACGATATT |
| mdm-miR171h-F | CCTGATTGAGCCGTGCCAAT |
| novel-miR397-RT | GTCGTATCGACTGCAGGGTCCGAGGTATTCGCAGTCGATACGACGCTGAC |
| novel-miR397-F | CGGCTGCTCACTTCTCTTCTGTC |
| miRNA common-R | ACTGCAGGGTCCGAGGTATT |

Table S2 TPM values of key miRNAs in sRNA-Seq

| miRNA name | Group | | |
| --- | --- | --- | --- |
|  | 818 | 818/818 | 818/419 |
| csi-miR395b-3p | 30.82±9.29 | 44.26±10.70 | 56.96±32.23 |
| novel_miR170 | 303.54±3.46 | 91.33±15.11 | 120.05±11.50 |
| vvi-miR535c | 1941.67±255.91 | 1086.35±244.31 | 819.71±235.21 |
| vvi-miR159c | 59073.31±2290.43 | 40237.65±4973.77 | 58284.73±3010.27 |
| nta-miR160a | 49.13±9.24 | 19.47±6.00 | 35.33±8.88 |
| mdm-miR171h | 204.03±22.67 | 55.17±11.36 | 38.31±6.71 |
| novel-miR397 | 86.96±24.33 | 38.25±5.78 | 18.20±3.79 |

Table S3 FPKM values of key mRNAs in the transcriptome

| Gene ID | Gene name | Group | | |
| --- | --- | --- | --- | --- |
|  |  | 818 | 818/818 | 818/419 |
| CSS0037571 | *CsAPS1* | 2.66±0.21 | 1.44±0.15 | 2.79±0.33 |
| CSS0023103 | *CsMYB30* | 11.38±1.00 | 20.27±3.49 | 10.67±0.30 |
| CSS0002903 | *CsDLO2* | 8.08±2.65 | 6.77±0.19 | 13.73±1.14 |
| CSS0034771 | *CsEP3* | 2.09±0.29 | 2.34±0.17 | 1.26±0.50 |
| CSS0037356 | *CsCYP78A9* | 1.81±0.70 | 5.28±1.85 | 3.97±0.07 |
| CSS0026887 | *CsCCR2* | 16.56±0.10 | 8.73±0.46 | 21.43±0.73 |
| CSS0000409 | *CsLCD* | 1.06±0.21 | 0.93±0.11 | 3.43±0.28 |
| CSS0015915 | *CsCCoAOMT* | 1.87±0.41 | 12.18±3.39 | 3.31±2.18 |
| CSS0000701 | *CsSCL6* | 4.13±0.31 | 4.43±0.95 | 7.86±1.66 |
| CSS0033521 | *CsLAX2* | 12.25±2.97 | 15.68±2.11 | 23.43±1.28 |

Table S3 Characteristics and sequences of newly identified novel miRNAs in grafted tea plants.

| Novel miRNA ID | Mature miRNA Sequence (5'→3') | Precursor Sequence (5'→3') | Precursor Length (nt) | Genomic Locus (Chr:start-end) |
| --- | --- | --- | --- | --- |
| novel-miR397 | ugcucacuucucuucugucagc | uuugucuguaguuucagaucuaacucauugccauaugauuuauucacacauguauacaauuugcuguguguauauggauguguuucugggcugaguaaucacucacucgcucaugagucaugacugaucagagggagugucugacagaagagagugagcacacuacaaacaauuguauaaaagauuauaccauuguggggugugugugcucacuucucuucugucagcuuucuaugugcccuucugguug | 250 | Chr2: 163,647,803-163,648,052 |
| novel-miR170 | uugcauacgcaccugaauugg | ggagcaaggcuugaaagggcaauggaggucgaugcucggugaaugguucgucgucgagacggagggaguaguguucucuucguggguuaagagagaaguagcggaagaguucguggcugugguggguuugggaaggagaagugggauugagguugaauuugagguggguugugaugaaaaucagggguggagaugagagagcaccaugauuugcauacgcaccugaauuggaggaggguuuugacgggga | 250 | Chr7: 65,288,077-65,288,326 |
